# Supplementary figures and images for: Facile Preparation of a Superhydrophobic iPP Microporous Membrane with Micron-Submicron Hierarchical Structures for Membrane Distillation
Source: Polymers (Basel). 2020 Apr 20;12(4):962. doi: 10.3390/polym12040962 (PMC7240455; doi:10.3390/polym12040962)

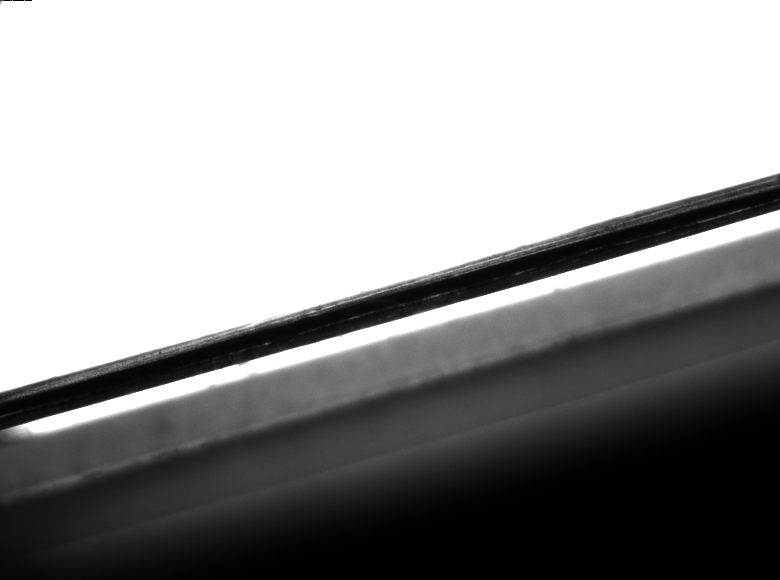

Supplement: Supplementary file 1 [file polymers-12-00962-s001.zip › Roll-off angle=15íπ.gif]

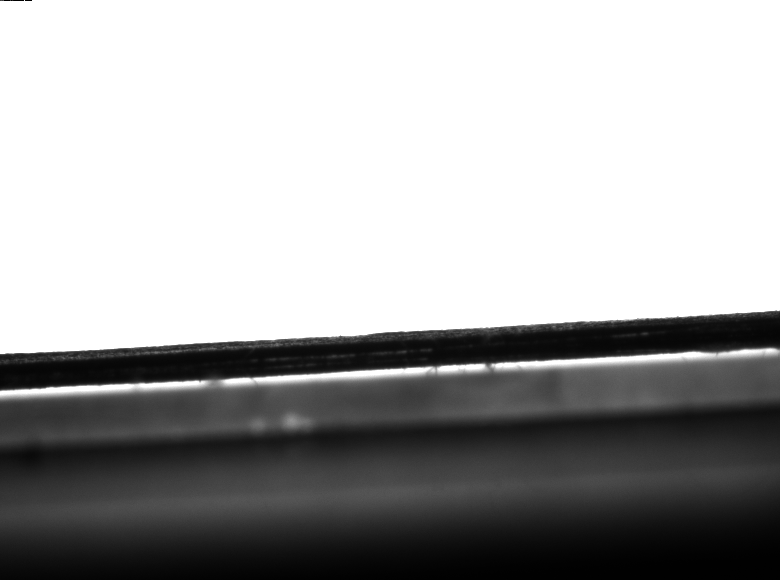

Supplement: Supplementary file 1 [file polymers-12-00962-s001.zip › Roll-off angle=2íπ.gif]

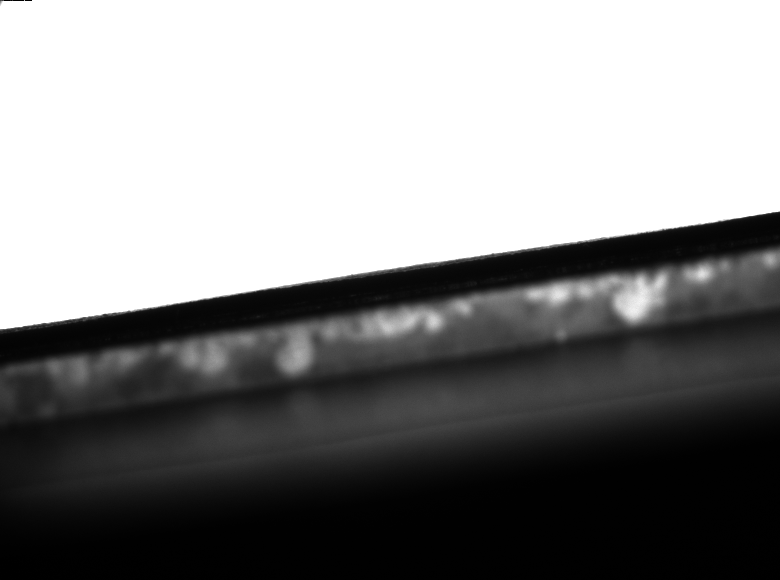

Supplement: Supplementary file 1 [file polymers-12-00962-s001.zip › Roll-off angle=8íπ.gif]

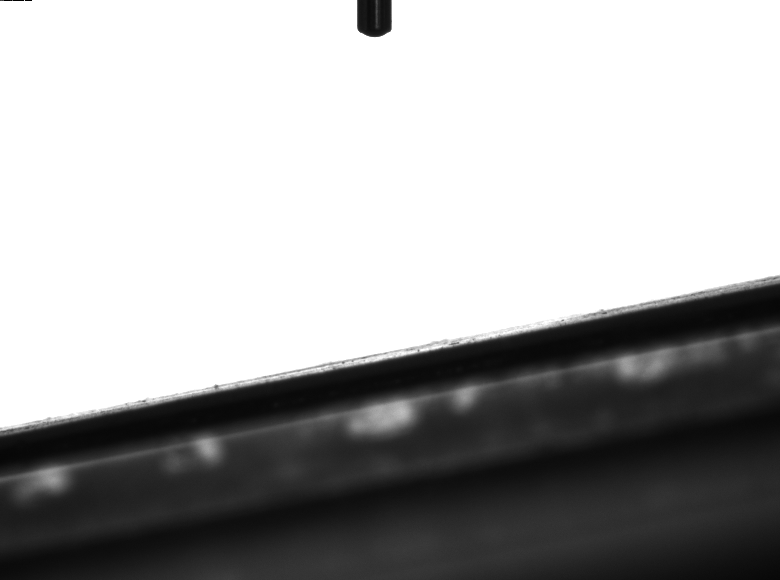

Supplement: Supplementary file 1 [file polymers-12-00962-s001.zip › Roll-off angle=10íπ.gif]
